# Health care utilisation under the 30-Baht Scheme among the urban poor in Mitrapap slum, Khon Kaen, Thailand: a cross-sectional study

**DOI:** 10.1186/1475-9276-6-11

**Published:** 2007-09-21

**Authors:** Sophie Coronini-Cronberg, Wongsa Laohasiriwong, Christian A Gericke

**Affiliations:** 1 Department of Public Health Administration, Faculty of Public Health, Khon Kaen University, Khon Kaen, Thailand; 2 Institute of Tropical Medicine and International Health, Charité Medical School, Humboldt and Free Universities, Berlin, Germany; 3 Chair in Public Health Policy, Discipline of Public Health, The University of Adelaide, Adelaide, Australia

## Abstract

**Background:**

In 2001, the Government of Thailand introduced a universal coverage scheme with the aim of ensuring equitable health care access for even the poorest citizens. For a flat user fee of 30 Baht per consultation, or for free for those falling into exemption categories, every scheme participant may access registered health services. The exemption categories include children under 12 years of age, senior citizens aged 60 years and over, the very poor, and volunteer health workers. The functioning of these exemption mechanisms and the effect of the scheme on health service utilisation among the poor is controversial.

**Methods:**

This cross-sectional study investigated the prevalence of 30-Baht Scheme registration and subsequent self-reported health service utilisation among an urban poor population in the Teparuk community within the Mitrapap slum in Khon Kaen city, northeastern Thailand. Furthermore, the effectiveness of the exemption mechanisms in reaching the very poor and the elderly was examined. Factors for users' choice of health facilities were identified.

**Results:**

Overall, the proportion of the Teparuk community enrolled with the 30-Baht Scheme was high at 86%, with over one quarter of these exempted from paying the consultation fee. User fee exemption was significantly more frequent among households with an above-poverty-line income (64.7%) compared to those below the poverty line (35.3%), χ^2 ^(df) = 5.251 (1); p-value = 0.018. In addition, one third of respondents over 60 years of age were found to be still paying user fees. Self-reported use of registered medical facilities in case of illness was stated to be predominantly due to the service being available through the scheme, with service quality not a chief consideration. Overall consumer satisfaction was high, especially among those not required to pay the 30 Baht user fee.

**Conclusion:**

Whilst the 30-Baht Scheme seems to cover most of the poor population of Mitrapap slum in Khon Kaen, the user fee exemption mechanism only works partially with regard to reaching the poorest and exempting senior citizens. Service utilisation and satisfaction are highest amongst those who are fee-exempt. Service quality was not an important factor influencing choice of health facility. Ways should be sought to improve the effectiveness of the current exemption mechanisms.

## Background

Thailand became the first transition country in Asia to introduce universal coverage for health care in 2002. This important step follows a longstanding commitment by the Thai Government to provide affordable health care to its population which is reflected by its high share of public expenditure for health care (61.6% in 2003) when compared to neighbouring transition countries [1]. When the Thai Rak Thai party claimed election victory in early 2001, it made good one of its main election promises – that of a national universal coverage scheme. Known colloquially as either the '30-Baht Scheme' or 'Gold Card Scheme', the new Government launched the initiative within three months of taking office [2]. As a result, the National Health Security Act was passed by parliament in November 2002, creating new institutions to regulate the quality and financial elements of the scheme. It preserved all benefit entitlements for members of the civil service and social security schemes but placed management of their financing with the National Health Security Office, which also manages the 30-Baht Scheme. The scheme is financed from general revenue.

The intention was to amalgamate the existing social welfare and voluntary health card schemes, and to extend coverage to the estimated 20–30% of the population of mostly informal sector workers that remained uninsured [3-6], thereby creating a more equitable system [7]. All individuals not currently enrolled in a health scheme and whose names were documented in house registrations, received a 'Gold Card', which has to be presented together with the patient's national identification card every time (s)he accesses health services [8]. The accessing of higher level health services occurs via referral from a registered primary health centre or hospital, except for emergency services which can be accessed at any public health service facility. A 30 Baht (US$0.92 or €0.68 in May 2007) co-payment is incurred for all consultations [9]. Children under 12 years of age, seniors over 60 years of age, volunteer health workers, and the very poor are exempt from this user fee. For the purposes of the scheme, the very poor were considered to be the beneficiaries of the previous Low-Income Card scheme and some other disadvantaged population subgroups.

The introduction of the policy apparently extended coverage to 18.5 million previously uninsured Thais [8]. Other estimates suggest insurance coverage increased from 71.0% of the national population in 2001 to 94.3% by 2004 [10]. Not only did the 30-Baht Scheme expand coverage, it also successfully maintained it for those previously covered by other types of health insurance [9].

Thailand's 30-Baht Scheme is just one example of programmes that have been implemented by governments around the world to improve the health of their poorest citizens. Yet, the existence of a pro-poor policy per se does not necessarily result in increased access to health care [11,12] and there is a growing recognition that these initiatives often do not reach those who need them most [13-16] or at least do not do so initially [17]. Instead, the Inverse Care Law [18] often prevails and better-off groups continue to benefit more than the poor from public health spending [19-21].

The evidence from Thailand to date is mixed. Studies indicate that since the introduction of the 30-Baht Scheme the poor have a very low burden of health care costs [22] and that Thailand's poor do indeed receive more benefits than the non-poor in outpatient care [10]. It has been reported that the proportion of households who were impoverished following medical treatment declined by about two thirds between 1992 and 2002 with at least some of these reductions attributed to the 30-Baht Scheme [23].

However, other research indicates that disparities persist. Suraratdecha et al. [9] found that 8.9% of respondents to a household survey conducted in three low-income Thai provinces were not covered by the 30-Baht Scheme, and that these were likely to have lower socioeconomic status. Other studies have shown that lower income groups often have access to lower quality health services when compared to more affluent population groups [24] as well as inequity in accessing and financing tertiary medical facilities which also tend to favour the rich [2,10]. In addition, despite the fact that the 30-Baht Scheme reduces health care expenditure for some households, there are households who opt out because of lengthy waiting times and lack of confidence in service quality [25]. Furthermore, specific population subgroups such as the urban poor are not represented in the literature, so little is known about the extent of their use of government health services.

People living in conditions of poverty lack a wide range of economic and other resources. Life expectancy is shorter and the prevalence of most diseases increases in line with the extent of poverty [26]. However, although we are aware of the detrimental effects of socioeconomic inequalities on health, they seem to be growing rather than shrinking globally [27-29].

The poverty threshold, or poverty line, is measured by a level of income or expenditure below which one cannot afford to purchase all the resources one requires to live and is therefore considered poor [30-32]. Since 2004, the official poverty line for Thailand has been set at 1,242 Baht per capita per month [33]. The percentage of the population living under the poverty line decreased substantially between 1988 and 2002: from 32.6% to 9.2%, with most of the decrease occurring before 1996 when the poverty incidence reached 11.4% [34]. However, Thailand's inequalities in terms of income distribution are higher than those in many other South East Asian countries. In 2002, the income disparity between the richest and poorest groups was found to be 13.4 fold [10]. The same year saw the poorest quintile of the population receive only about 12% of government health expenditure while the richest quintile received three times as much [35]. In addition, research suggests that very few of the ultra poor receive any assistance despite the government's numerous social assistance schemes [36].

The rich-poor divide and poverty incidence rate is characteristically smaller in urban than in rural areas. This also applies to Thailand [37], where 19.7% are classified as poor in rural areas, compared to 6.7% of the urban Thai population [10]. The urban population in Asia is forecast to keep growing in coming years. In 1999, 36.2% of the Asian population was urbanised with an urban growth rate of approximately 3.77% per year. By 2030, Asia, together with Africa, will have higher numbers of urban dwellers than any other major area of the world [38]. Despite urban dwellers generally being considered better off, UN-HABITAT [39] disputes the assumption that poor urban populations are healthier. Instead, it finds that slum-dwellers pay a so-called 'urban penalty', meaning they are as badly or worse off than their rural counterparts, and are more likely to experience hunger and disease.

Thailand's continuing rapid urbanisation is strongly associated with economic migration from rural areas. By the end of 2006, the city-dwelling population was forecast to have reached 42–43% of the total population [37]. This predicted continued swelling of cities is likely to be accompanied by a growth in slums. Although the Global UN-HABITAT scorecard rates Thailand as having slowed slum growth in the last 15 years, the kingdom still has a significant population of urban poor. In 2001, 20% of Thailand's 64 million population was urban, with 2% living in slums [38]. This puts the figure significantly lower than other research. Boonyabancha [40] finds Thailand had some 5,500 low-income urban communities, containing 8.25 million inhabitants (12.9% of the population), living in poor quality and often insecure housing in 2003. Despite its size, access and affordability of health care for this population has not been studied in detail.

The Northeastern province, Isaan, of which Khon Kaen is the capital, has the highest poverty rates in Thailand [32]. Khon Kaen has an estimated population of 150,000 and a number of large slum districts. The objective of this study was to examine the prevalence of self-reported registration with the 30-Baht Scheme and subsequent health services utilisation through the 30-Baht Scheme by inhabitants of Mitrapap slum in Khon Kaen, Thailand. Little formal information had been gathered about this population to date, but it was believed inhabitants commonly suffer from poverty, poor housing consisting of non-durable materials and inadequate piped water supply. Furthermore the area is prone to seasonal flooding. This is consistent with existing literature profiles of Asia's – including Thailand's – urban poor [40-42].

This study sought to ascertain whether Mitrapap's urban poor population is adequately covered by the 30-Baht Scheme. More specifically, it looked to: ascertain the health insurance status of the study population and describe the characteristics of who is enrolled with the 30-Baht Scheme; assess whether households registered with the 30-Baht Scheme use it to access health services; and seek to explain why the study population does or does not access health services through the 30-Baht Scheme. Furthermore, the effectiveness of the current user fee exemption mechanisms for the very poor and the elderly was examined.

## Methods

A cross-sectional study was conducted in July 2006 in two villages from the largest slum settlement in Khon Kaen city, Mitrapap. It consists of four separate communities: Teparuk, Makham, Chumchon Lung Soon Ratchagan, and Rod Fai. Chumchon Lung Soon Ratchagan and Rod Fai were considered too dangerous for fieldwork due to violent resident protests against a proposed slum-dweller eviction programme. Of the two remaining communities, Makham and Teparuk, both were considered suitable. Teparuk was chosen because there was a strong existing relationship between researchers and community leaders, better enabling access to the community.

Teparuk community consists of three villages, Teparuk 2, Teparuk 3 and Teparuk 4. Teparuk 2 is separated from the other villages by a large road and consists of 25 households. The pre-test was carried out here in the week preceding actual data collection, the results from which have not been included in this study. Teparuk 3 and Teparuk 4 are adjacent villages, situated on a piece of land approximately 50 m wide, that is bordered by a road on one side and a railway line on the other. The study sampled as close to 100% of the resident population of Teparuk 3 and 4 as possible. This survey is based on the responses of 72 households of an estimated 100 households. No data on the socioeconomic status of the non-included households was available.

A structured questionnaire was developed to gather information from respondents. Information from other, similar questionnaires was used as a guideline. In line with other slum populations, the average education level of Teparuk inhabitants was assumed to be low and it was therefore decided to use interviewer-applied questionnaires. The tool was proofed by three senior academics from Khon Kaen University. Once completed, the questionnaire was translated from English into Thai by one of the authors (WL) and cross-checked by colleagues.

Prior to fieldwork, village volunteer health workers, community leaders and health centre staff were contacted to co-ordinate visits of the interviewers with the heads or representatives of the households. The interviewers consisted of eight final-year undergraduate students from the Faculty of Public Health, Khon Kaen University. All had previous field survey and interviewing experience, including conducting questionnaire-led interviews. Prior to the pre-test, a detailed training session was held. A short refresher briefing was arranged just before the start of each fieldwork session.

The fieldwork was conducted in two separate sessions in mid-July 2006, at two different times of day. One individual from each house was approached for interview and his/her responses were used as a proxy for household health services utilisation. For the purposes of this research a household was defined as: individuals living in the same housing unit, who may or may not be relatives but who share financial and other resources. If the selected individual could not communicate effectively with the interviewer, was under 15 years of age or refused to participate, the researcher sought to select another suitable individual residing in that household. If individuals were busy or not available, interviewers either tried to agree another appointment time or made a note to return to the house at a later time the same day, or at the next field visit (if applicable). Also, if during the course of the interview the researcher established that other financially separate groups shared the house, the researcher sought to interview a representative from each group.

Initially, it was planned to select households in Teparuk 3 and Teparuk 4 by randomised proportional sampling. Due to heavy rainfall and subsequent flooding in parts of Mitrapap just prior to the fieldwork, many households were forced to move. The remaining estimated population was too small to sample, so it was decided to undertake systematic sampling and try to capture one representative of each household in both of the Teparuk villages. In addition, the start of the rice-planting season may have precipitated seasonal migration to rural areas, something that particularly affects those engaged in unskilled labour occupations. As this tends to be the poorest stratum of a poor population, this survey may not have captured the poorest members of Mitrapap community and may therefore not be completely representative of other urban slums in Thailand.

Interviewers obtained consent from all participants. Information was collected on the socioeconomic and demographic status of the respondents, their health insurance status, their perception of and utilisation of health services, and their current health status. They were also asked to report diseases they had had in the month preceding the survey. For those who had been sick, information was collected on whether and where treatment was sought, how the disease was treated and reasons for choosing a particular care provider.

Both inter- and intra-questionnaire variation was checked. Responses from each interview were recorded on the questionnaire forms, and the responses were coded. Collected data were double-entered into an MS Excel spreadsheet and then imported into and analysed using the software package SPSS 11.5. Frequencies, percentages and, where applicable, means, standard deviations, medians and ranges were calculated, and tests of significance were carried out.

One of the inherent problems with populations engaged in the informal sector is the lack of data on their financial status. This study therefore had to rely on self-reported assessments of income. However, by surveying a population with which researchers from the University have a good relationship it is believed that inaccuracies have been kept to a minimum.

Since respondents cannot be forced to take part in a survey such as this, self-selection bias is always a potential problem, especially when trying to reach the poor [43]. By informing the health centre and community leaders of this research, it was hoped that participation would be maximised. Only six people refused to participate. Approximately fifteen households did not have any eligible members at home on either day. There was one instance where a section of the slum was deemed unsafe for the interviewers to enter, due to the threat of violence. This section, containing an estimated five houses had to be left out.

## Results

All surveyed individuals were Thai citizens, possessed a Thai identity card and, with one exception, the houses of those surveyed were all registered with the authorities. The overwhelming majority (91.7% of respondents) defined themselves as literate. For the purposes of this research, 'literacy' was defined as being able to read and write Thai. However, 75% of respondents had only received primary school education or less.

### Health insurance coverage

The proportion of households registered with the 30-Baht Scheme was 86.1%, whilst just two households had no health insurance at all (Table 1). Households where where respondents had attended secondary school or had a vocational/bachelors degree compared to respondents with only primary school education were significantly more often registered with the 30-Baht Scheme (χ^2 ^(df) = 10.932 (2); p-value = 0.004). With one exception, all households were registered at facilities in Khon Kaen. 54.1% were enrolled only at the regional hospital, while 31% were registered with both a primary care unit and a hospital. Only 27.4% of households were exempted from paying the 30 Baht user fee when accessing health services at registered facilities.

**Table 1:** Health insurance coverage

	**Health insurance**
	
**Households (n = 72)**	**Number (%)**
30-Baht Scheme	62 (86.1)
Other health insurance	8 (11.2)
Uninsured	2 (2.7)

### Socio-economic profile of households covered by the 30-Baht Scheme and user fee exemptions

The median household income was 5,750 Baht, ranging from 550 to 30,000 Baht. 61.3% of households covered by the 30-Baht Scheme were below the national poverty line of 1,242 Baht per capita per month (Table 2). All households below the poverty line but none above stated that they do not generate sufficient income to cover their total necessary monthly cost. 53.2% of households stated that they are in financial debt. Only 19.4% of households had any cash savings.

**Table 2:** Socioeconomic profile of households enrolled with the 30-Baht Scheme

	**30-Baht user fee**		
	**Pay**	**Exempt**	**Total**
			
**Households (n = 62)**	**Number (%)**	**Number (%)**	**Number (%)**
**Poverty line (≤THB 1,242/head/mth)**			
Above	13 (54.2)	11 (45.8)	24 (38.7)
Below	32 (84.2)	6 (15.8)	38 (61.3)
**Overall household financial status**			
Savings	11 (91.7)	1 (8.3)	12 (19.4)
Debts	25 (75.8)	8 (24.2)	33 (53.2)
Neither savings nor debts	9 (52.9)	8 (47.1)	17 (27.4)
**Income sufficient to cover costs**			
Yes	20 (83.3)	4 (16.7)	24 (38.7)
No	25 (65.8)	13 (34.2)	38 (61.3)

45.8% of those above the poverty line were exempt from paying the user fee compared to only 15.8% of those below the poverty line (Table 2). Put another way, 64.7% of households not paying user fees are above the poverty threshold. The difference in user fee exemption between those above and below the national poverty line was statistically significant: χ^2 ^(df) = 5.251 (1); p-value = 0.018. Using the median household income of 5,750 Baht as the threshold yielded a comparable result: χ^2 ^(df) = 5.795 (1); p-value = 0.016. Of those with an insufficient monthly income to cover all necessary household costs, 65.8% were still required to pay 30 Baht to access facilities, as were 75.8% of those with debts.

### Subjective health status and reported illness

43.1% of households rated their overall health as fair to very poor (Table 3). Hypertension and peptic ulcers were the most common types of physician-diagnosed chronic diseases present in the sample. In the four weeks prior to the survey, 41.7% of all surveyed households recalled experiencing illness, and 80% of these sought medical treatment.

**Table 3:** Subjective health status

	**30-Baht Scheme**		
	**Enrolled**	**Not enrolled**	**Total**
			
	**Number (%)**	**Number (%)**	**Number (%)**
**Self-perceived health status**			
≥Good	33 (53.2)	8 (80.0)	41 (56.9)
Fair	15 (24.2)	0 (0.0)	15 (20.8)
≤Poor	14 (22.6)	2 (20.0)	16 (22.2)
Total	62 (100.0)	10 (100.0)	72 (100.0)
**Prescence of chronic disease**			
Yes	19 (30.6)	1 (10.0)	72 (100.0)

Of those registered with the 30-Baht Scheme, 46.8% classified their health as fair to very poor, compared to 19.5% of those not covered by the 30-Baht Scheme. A positive association between self-perceived health status and enrolment with the 30-Baht Scheme was found: χ^2 ^(df) = 9.629(2); p-value = 0.008. There was also a positive association between the presence of chronic disease and 30-Baht Scheme coverage: χ^2 ^(df) = 4.704(1); p-value = 0.030 (Table 3).

### Utilisation of registered health services and user satisfaction

Respondents were asked to rank their three most-consulted health service providers in order of preference. Tertiary care was the highest scoring option, followed by primary health care units and then pharmacies. While choice was most often determined by whether the health facility was registered with the 30-Baht Scheme, proximity to home and the existence of good transport links were also important reasons for the choice of provider.

Overall, utilisation seemed to have increased since the introduction of the 30-Baht Scheme. 52.5% of registered households claimed to have increased their use of health services since the introduction of the scheme (Table 4). 89.8% stated they now had better access to health services than before. The majority of patients with a Gold Card always accessed medical help at registered facilities (80.3%). 81.2% of those covered by the 30-Baht Scheme recalled an illness episode of a household member in the four weeks preceding the survey, 84% of these sought treatment and all consulted with 30-Baht registered facilities. Of those exempted from paying the user fee, all sought treatment. The most commonly cited reason (45.8% of households) for selecting to seek treatment at a registered facility was the fact that it was registered with the scheme.

**Table 4:** Utilisation of 30-Baht Scheme registered health services and user satisfaction

	**30-Baht user fee**		
	**Pay**	**Exempt**	**Total**
			
**Households (n = 61)#**	**Number (%)**	**Number (%)**	**Number (%)**
**Use of registered facility**			
Always	34 (77.3)	15 (88.2)	49 (80.3)
Often	3 (6.8)	2 (11.8)	5 (8.2)
Sometimes	5 (11.4)	0 (0.0)	5 (8.2)
Never	2 (4.5)	0 (0.0)	2 (3.3)
Households (n = 59)*			
			
**Health care access**			
≥better than before Gold Card	38 (90.5)	15 (88.2)	53 (89.8)
Same as before Gold Card	4 (9.5)	2 (11.8)	6 (10.2)
**Utilisation of health services**			
Increased since Gold Card	21 (50.0)	10 (58.8)	31 (52.5)
Same or worse since Gold Card	21 (50.0)	7 (41.2)	28 (47.5)
**30-Baht Scheme satisfaction**			
≥Satisfied	41 (97.6)	17 (100.0)	58 (98.3)
≤Unsatisfied	1 (2.4)	0 (0.0)	1 (1.7)

**# **= one respondent household was registered outside Khon Kaen municipality

* = three non-respondents

Overall, satisfaction with the scheme was found to be very high at 98.3% (Table 4). However, approximately one third of those seeking health care using the Gold Card cited opening hours of the health facility (36.1%) and loss of income (29.5%) as problems. Amongst those respondents required to pay user fees, opening hours and loss of income presented difficulties for 68.2% and 77.8%, respectively. Cost of treatment, disrespectful staff and lack of services available for particular health problem were issues more often cited by respondents who were required to pay the 30 Baht user fee.

## Discussion

With under 3% of Terapuk 3 and 4's population uninsured, this survey suggests national estimates of approximately 94% health insurance coverage [10] across the Thai population are being achieved, even among Khon Kaen's urban poor.

One of the key principles behind public health insurance schemes is to protect an individual's income and assets from debilitating financial problems associated with expensive medical care [4]. The results suggest this population is a suitable target group for a pro-poor initiative such as universal health care coverage. Four out of five households have no savings, 70.8% reported an average monthly household income 60% or less than the national average (13,418 Baht in 2006), and 62.5% fall under the national poverty line. The results also highlight a positive association between presence of chronic disease and being exempted from paying the 30 Baht consultation fee. This suggests the system has a positive impact on equity of access and finance, since the link between having a chronic disease and poverty, or wealth-depletion, is well described [44,45].

Existing literature suggests that both opportunity costs and low levels of education still inhibit the poor from accessing health care services in Thailand [9]. However, this conclusion is not supported by the survey findings. Although three quarters of residents living in Mitrapap Community have received primary education or less, the vast majority (96.7%) of those registered said they most frequently consult 30-Baht registered services, both for elective and emergency care. Furthermore, 90% feel the 30-Baht Scheme improved their access to health services. In order to cross-reference this, respondents were asked to recall any episodes of illness in the four weeks prior to the survey and to give details of any treatment they accessed. Of those who have Gold Cards and sought medical advice, all consulted services at 30-Baht registered facilities. Two thirds confirmed that the fact the facility is part of the scheme was a reason for choosing to access care at that particular centre.

Initiatives such as the Gold Card Scheme inherently assume that financial cost of access is the main barrier to the poor accessing services. Although the majority (89.8%) of households stated their access to health services had increased since the introduction of the 30-Baht Scheme, self-reported utilisation rates increased among only half (52.5%) of those surveyed. Whilst registration status of a health centre was an important factor influencing choice, options pertaining to quality of service were not regularly selected suggesting cost was the main motivator. Existing literature, however, suggests removing financial barriers to health services may not be sufficient to provide access for the poor if other factors, for example the quality of services, is deemed insufficient [46]. There are also opportunity costs to be considered [9], such as cost and availability of transport or loss of earnings. One third of households questioned cited the opening hours of health facilities and also loss of income as issues restricting their access. In addition, loss of income and inconvenient opening hours posed a greater problem for those who have to pay the user fee.

This might be indicative of the 30-Baht Scheme not going far enough in enabling equitable access, since it is limited to easing financial constraints. Studies into Thai health service quality, including clinic opening hours, remain rare [24]. However, where they do exist the findings suggest considerations such as service quality and geographical access also need to be taken into account when trying to improve access to health services for the poor.

The results show that 84.4% of inhabitants with monthly incomes under the national poverty line are covered by the scheme, as are 80% of those without savings and approximately three out of five who feel the household income is insufficient to cover necessary monthly costs.

Of those enrolled with the 30-Baht Scheme, just over a quarter are exempt from paying the user fees. Exemption from user charges is one way in which access for the poorest of the poor is often thought to be enabled. However, in line with other studies [47,48], the survey results indicate that the fee-waiver mechanism may not exempt all those it should. We found that the exemption mechanism actually favoured households with an above-poverty-line income (64.7% user fee exemptions) compared to those below the poverty line (35.3% exemptions). Likewise 90% of those with an insufficient monthly income to cover necessary costs are enrolled with the 30-Baht Scheme, but two thirds (65.8%) of these still have to pay the user charge. Similarly only 24.2% of those with debts are able to access the health system for free. Since one of the central aims of the Thai universal coverage scheme is to waive the 30 Baht user fee for the poorest segment of the population, these findings indicate that the current exemption mechanism is partially failing this objective.

One problem of using a poverty line is that it assumes the poor are a static group. In reality, however, people move in and out of poverty meaning there is a subgroup at risk of poverty [49,50]. Perhaps financial status among the surveyed population is changing more quickly than the exemption process can keep up with and thereby some of those that should be exempt from paying user fees are not. Another reason may be the difficulty of quantifying income for groups where many work in the informal sector [32]. Similarly, assessing poverty is not just linked to financial resources [49]: urban Thai households tend to save using tangible assets such as jewellery [51] which can easily be pawned if cash is needed. Since financial status may not be indicative of assets held, community elders decide who is poor and in consequence who should be exempted from the 30 Baht fee. A study from neighbouring Cambodia concludes community assessment is a feasible and effective method [52]. By contrast, Walt [53] argues that although the assessment may be accurate, the final decision of who to include may be influenced by social phenomena. Community-assessed payment exemptions therefore cannot ignore the socio-cultural and political realities of communities [43]. This could influence who is exempted – both positively and negatively.

Another concern is that 31.6% of elderly citizens enrolled with the 30-Baht Scheme are still paying user fees in spite of the automatic fee exemption for all those over 60 years of age [54]. This suggests that there is either a system failure, or that patients are not sufficiently well-informed about how to obtain the exemption.

Official identification documents, or a house certificate, and Thai citizenship are pre-requisites for anyone wishing to apply for a Gold Card [55]. The card is issued at the municipality district of residence, where it can only be used at a designated primary care unit and hospital. Anyone moving to a different district cannot utilise health services there without going through a re-application procedure [2]. It was therefore anticipated that the prevalence of registration in Mitrapap would be low, since slum settlement populations tend to be unstable, conditions that could prevent the poorest from gaining access to health care. However, no respondent, even those that considered themselves illiterate, lacked the necessary documents. This may be due to the population not being a transient one: the median number of years lived in Teparuk 3 and Teparuk 4 was 15 years in this study.

## Conclusion

At 86%, the overall coverage of the 30-Baht Scheme looks impressive among the population of Mitrapap slum in Khon Kaen. The vast majority of households enrolled in the 30-Baht Scheme say their access to health services has improved since the introduction of the scheme. Furthermore, most state that they are satisfied with the health services they receive.

However, a number of equity issues persist. The current user fee exemption mechanism fails to distinguish the poor from the poorest and actually favours those with an income above the national poverty line compared to those below. It also fails to exempt all those over 60 years of age – an exemption that should be automatic and easy to administer as all patients have to present the Gold Card and their Thai identity card whenever they seek health care at a registered facility. In addition, the main motivation for choosing a 30-Baht Scheme registered service is cost, with little concern for the quality of services dispensed. Despite the apparent success of the scheme in decreasing direct health care costs, indirect costs such as loss of income are still a problem for about a third of households.

This research highlights a number of areas for further investigation, notably how best to define who is 'poor' and subsequently to improve exemption mechanisms to better target this group. It would also be of value to ascertain why those aged above 60 years are not automatically excluded from paying user fees.

Despite Gold Card holders overwhelmingly stating that they are happy with the services they receive, this may not necessarily be a true indicator. Lack of education [56], power imbalances [57] or social concerns may be playing a role in making people unwilling to speak openly. A deeper qualitative analysis into peoples' views could corroborate these findings.

The abolition of the very popular 30-Baht Scheme at the end of 2006 by General Sonthi Boonyaratglin's Government after the ousting of Prime Minister Thaksin who had introduced the scheme in the first place was motivated by political reasons and not because it was seen as a failure.

## Competing interests

The author(s) declare that they have no competing interests.

## Authors' contributions

All three authors conceived and designed the study. SCC conducted the survey, analysed the data and wrote the first draft of the manuscript. WL and CAG critically revised the manuscript and contributed significantly to the final version of the manuscript. All authors read and approved the final manuscript.
